# Bulked-Segregant Analysis Coupled to Whole Genome Sequencing (BSA-Seq) for Rapid Gene Cloning in Maize

**DOI:** 10.1534/g3.118.200499

**Published:** 2018-09-07

**Authors:** Harry Klein, Yuguo Xiao, Phillip A. Conklin, Rajanikanth Govindarajulu, Jacob A. Kelly, Michael J. Scanlon, Clinton J. Whipple, Madelaine Bartlett

**Affiliations:** * Plant Biology Graduate Program and Biology Department, University of Massachusetts Amherst, Amherst, MA 01003; † Department of Biology, Brigham Young University, 4102 LSB, Provo, UT 84602; ‡ Department of Plant Biology, Cornell University, Ithaca, NY 14853; § Department of Biology, West Virginia University, Morgantown, WV 26506

**Keywords:** forward genetics, maize genetics, mutant genes, bulked-segregant analysis, next generation sequencing

## Abstract

Forward genetics remains a powerful method for revealing the genes underpinning organismal form and function, and for revealing how these genes are tied together in gene networks. In maize, forward genetics has been tremendously successful, but the size and complexity of the maize genome made identifying mutant genes an often arduous process with traditional methods. The next generation sequencing revolution has allowed for the gene cloning process to be significantly accelerated in many organisms, even when genomes are large and complex. Here, we describe a bulked-segregant analysis sequencing (BSA-Seq) protocol for cloning mutant genes in maize. Our simple strategy can be used to quickly identify a mapping interval and candidate single nucleotide polymorphisms (SNPs) from whole genome sequencing of pooled F2 individuals. We employed this strategy to identify *narrow odd dwarf* as an enhancer of *teosinte branched1*, and to identify a new allele of *defective kernel1*. Our method provides a quick, simple way to clone genes in maize.

Forward genetics remains a powerful way to ‘ask the plant’ which genes matter for a particular trait or phenotype (Mueller 2006). Because forward genetics relies on random mutagenesis, it presents an unbiased method for identifying novel genes that act in particular pathways. While forward genetic screens can reveal new genes, they can also reveal novel functions for known genes; providing a richer, more deeply nuanced view of gene function essential for a true understanding of how genes and gene networks contribute to building an organism (Nawy *et al.* 2010; Gallavotti *et al.* 2010; Vlad *et al.* 2014; Gillmor *et al.* 2016). After random mutagenesis, mutant genes are most often identified through linkage mapping.

Linkage mapping, using a combination of bulked-segregant analysis (BSA) and fine mapping, has been very successful for cloning maize genes (Gallavotti and Whipple 2015). The process starts when a mutant of interest is crossed to a wild-type individual in a contrasting genetic background. The resulting F1 individuals are selfed or backcrossed, and mutants are identified in the F2 or backcross population. These mutants can be used to identify a region of increased homozygosity in a chromosomal region physically linked to the lesion causing the mutant phenotype. This region of increased homozygosity can be rapidly detected using bulked-segregant analysis (Michelmore *et al.* 1991).

In bulked-segregant analysis, pools of wild-type and mutant individuals are genotyped at markers spread across the genome. In chromosomal regions not linked to the mutant lesion, markers will be segregating according to typical 1:2:1 (or 1:1 in backcross) segregation ratios. In a pooled BSA sample, these unlinked markers will all be genotypically heterozygous. Linked markers will be homozygous for the mutant parent genotype, unless F1 recombination has happened between the mutant lesion and a particular marker. A chromosomal region enriched for these linked markers represents a likely location for the mutant gene under study, and the coordinates of these linked markers define an initial mapping interval. Once this initial mapping interval is identified, F1 recombination is again used to place recombination breakpoints in individual mutants, and thus narrow the mapping interval using fine mapping (Gallavotti and Whipple 2015). The fine mapping process is often time consuming, and identifying causative lesions and mutant genes can take years.

The advent of next-generation sequencing (NGS) means that BSA can be used to very quickly identify a very small mapping interval, and even the causative lesion, without fine mapping. In BSA-Seq, whole genome shotgun sequencing using NGS can be used to quickly genotype mutant *vs.* non mutant BSA pools at many thousands of markers spread across the genome. These BSA-Seq data can reveal the genomic interval that contains the mutant gene of interest, and with enough coverage, the lesion itself (Zou *et al.* 2016). Since random mutagenesis produces lesions throughout the genome, and since NGS does not depend on established genotyping assays, BSA-Seq can be used to identify a chromosomal region using just the polymorphisms induced by mutagenesis. In this case, the contrasting genetic background used for the BSA can simply be that of an unmutagenized parent. This modification to BSA-Seq has been called MutMap (Abe *et al.* 2012). BSA-Seq (and MutMap) has been used to identify mutant loci in *Arabidopsis thaliana*, soybean, barley, *Mimulus*, rice, sorghum, and *Brachypodium distachyon* (Schneeberger and Weigel 2011; Abe *et al.* 2012; Mascher *et al.* 2014; Woods *et al.* 2014; Addo-Quaye *et al.* 2017; Ding *et al.* 2017; Song *et al.* 2017; Jiao *et al.* 2018). In maize, BSA coupled to RNA-Seq (BSR-Seq) has been used successfully to clone mutant genes (Liu *et al.* 2012; Li *et al.* 2013; Nestler *et al.* 2014; Tang *et al.* 2014). Conventional BSA-Seq has been used to identify genomic regions underlying variation in flowering time and plant height QTL in a maize population (Haase *et al.* 2015), but cloning mutant genes using BSA-Seq is not yet routine in maize.

Here, we report a user-friendly protocol for cloning mutant maize genes using BSA-Seq. We used this protocol to clone two genes recovered from EMS mutagenesis screens. We used conventional BSA-Seq in the case of one gene, and MutMap in the other. We identified *narrow odd dwarf* (*nod*) as an enhancer of *teosinte branched1* (*tb1*) using conventional BSA-Seq, and identified a seedling lethal allele of *defective kernel1* (*dek1*) using MutMap (Doebley *et al.* 1997; Lid *et al.* 2002; Becraft *et al.* 2002; Rosa *et al.* 2017). In addition, we used our *tb1* enhancer data to design insertion-deletion (indel) markers for downstream fine mapping. This fine mapping to reduce the recombination interval is critical in cases where only a single allele for a particular mutant exists, and in cases where no clear candidate lesion is identified. Our method provides a quick, easy method for cloning mutant genes in maize.

## Methods

### Plant material and isolation of mutants

**Figure 1:**
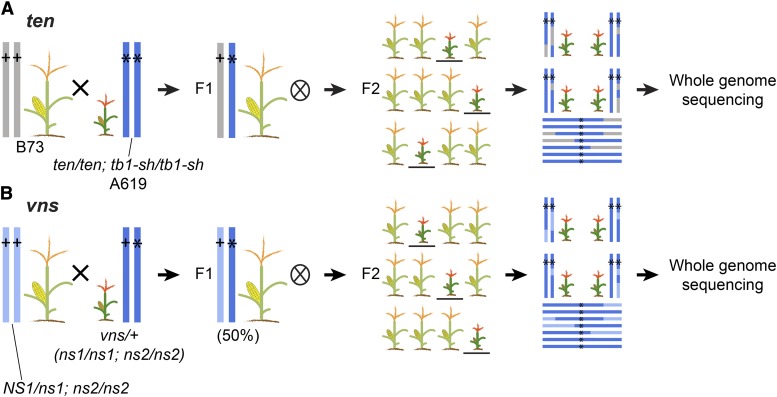
Crossing scheme to generate *ten* and *vns* mapping populations. (A) For *ten*, we crossed a homozygous *ten tb1-sh* double mutant (in A619) to a wild-type individual of a widely divergent genotype (B73), then selfed many plants in the resulting F1. We selected *ten* mutants in the F2 generation, regardless of the *tb1* phenotype. We pooled tissue from 101 of these mutants into a single DNA extraction and a single library for whole genome shotgun sequencing. (B) For *vns*, we backcrossed a plant heterozygous at *vns* to a wild-type individual of the unmutagenized parental genotype. We selfed many individuals in the resulting F1 (50% heterozygous at *vns*). We selected 9 *vns* mutants in the F2 generation, regardless of the *ns* phenotype, and pooled tissue into a single DNA extraction and NGS library.

To identify enhancers and suppressors of the *tb1* (Doebley *et al.* 1997) and *narrow sheath* (*ns)* (Scanlon *et al.* 1996) phenotypes, we performed EMS mutagenesis screens. *tb1* encodes a TCP transcription factor with a well characterized role in suppressing axillary branching or tillering (Doebley *et al.* 1997). The *ns* loci regulate the initiation of lateral cells in the maize shoot apical meristem. *ns1* and *ns2* encode duplicate homeodomain transcription factors, homologous to *PRESSED FLOWER* in *Arabidopsis thaliana* (Nardmann *et al.* 2004). We mutagenized pollen homozygous for a weak allele of *tb1* in the A619 inbred (*tb1-sh*), and *ns* in an unknown background obtained from Pioneer Hi-Bred Intl. (*ns1;ns2*), using established protocols (Neuffer *et al.* 1997). After mutagenesis, M1 progeny were selfed to generate M2 populations, where we identified the mutants *tb1 enhancer* (*ten*) and *very narrow sheath* (*vns*). *ten*, which arose in the A619 genetic background, was crossed to the B73 genetic background and then selfed to generate an F2 mapping population (Figure 1a). *vns* is seedling-lethal. Therefore a wild-type sibling, heterozygous at *vns*, was backcrossed to a *ns1* heterozygote fixed for *ns2 (ns1/NS1;ns2/ns2)*, and the progeny selfed to generate a mapping population (Figure 1b).

For BSA-seq, tissue samples were collected from 101 *ten* mutant individuals, and 9 *vns* mutant individuals in the mapping populations. We used this small number of *vns* individuals because that was what we had available from a small mapping population. Mutants in the F2 mapping populations were sampled independent of their genotypes at *tb1* or *ns1* (*i.e.*, neither *ten* nor *vns* was dependent on *tb1* or *ns*, respectively). Because the *vns* genetic background is unknown, we also collected tissue samples for 9 wild-type siblings in the *vns* mapping population and for the parents of the EMS mutagenesis screen. To characterize the genetic interaction between *tb1* and *ten*, we counted tillers in families segregating *tb1-sh* and *ten*.

### DNA extractions and NGS sequencing

Tissue samples were pooled by hole punching each leaf twice to ensure equal representation of individuals. We extracted DNA from the pools of tissue using a CTAB method (Gallavotti and Whipple 2015). Libraries were prepared using the TruSeq DNA sample prep kit according to the manufacturer’s instructions (Illumina). We sequenced the *ten* mutant pool on an Illumina Hi-Seq 2500 at Brigham Young University. Sequenced reads were 125bp long with paired-ends. We sequenced *vns* mutants, *vns* wild-type siblings, and unmutagenized parents on an Illumina Hi-Seq 2500 at Cornell University. Sequenced reads from *vns* were 150bp long with paired ends. Different sequencing platforms were used because of differing availability at our respective institutions.

### NGS quality control and read alignment

**Figure 2:**
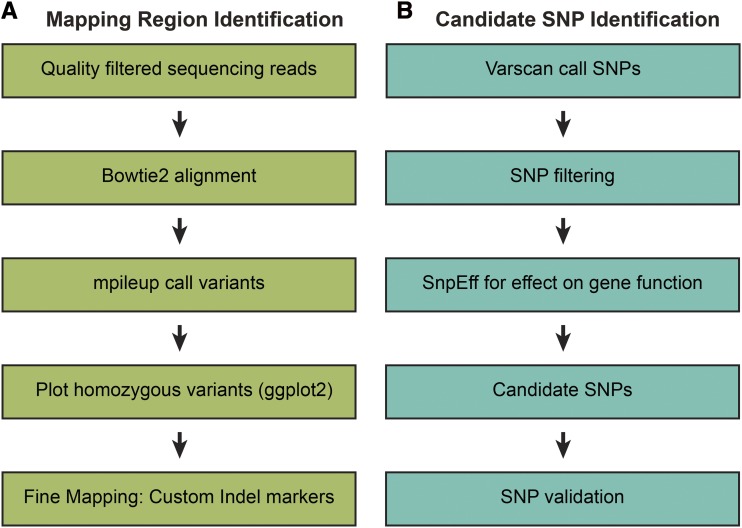
A simplified overview of our BSA-Seq pipeline. (A) We analyzed our NGS data in Galaxy and in R to identify chromosomal regions that contained the mutant genes. (B) SNP filtering to identify candidate (EMS) SNPs.

We used the genomic tools hosted on Galaxy web portal to process our Next-Gen sequencing data (Afgan *et al.* 2016). An overview of our process is shown in Figure 2. We used FastQC (v. 0.69) to determine the quality of our sequencing data, and established a PHRED quality cutoff of 20 based on this analysis (Ewing *et al.* 1998; Andrews 2014). We used FASTQ Groomer (v. 1.1.1) to convert our FASTQ files to Sanger format for input into downstream Galaxy tools (Blankenberg *et al.* 2010). Trimmomatic (v. 0.36.3) was used for sliding window trimming averaged across 4 base pairs with average quality >20 and for adapter sequence removal (Bolger *et al.* 2014). The Galaxy tool cat, which concatenates datasets tail-to-head (cat) (v. 0.1.0) was used to join sequencing files together if data were split from multiple Illumina sequencing runs (Afgan *et al*. 2018). We used Bowtie2 (v. 2.3.2.2) to align sequencing reads to the B73 reference genome version 4, release 56 and to generate an index (Langmead and Salzberg 2012; Jiao *et al.* 2017). Read alignment was assessed with Mtools Flagstat (v. 2.0) (Li *et al.* 2009).

### Variant calling

SAMtools mpileup (v. 2.1.3) was used to output variants from the indexed bam file into pileup or variant call format (vcf) files (Li *et al.* 2009). Pileup files were generated for identification of the mapping region and vcf files were generated for identification of candidate SNPs. To filter variant files for mapping region identification, we used SAMtools filter pileup (v. 1.0.2), a custom Galaxy tool, to filter based on read coverage and quality (Li *et al.* 2009). We filtered out non-variant positions, and focused only on variant positions with coverage of 8 or more reads, each with a quality score of 20 or more. To generate vcf files for candidate SNP identification, we used Varscan (v. 0.1). We used a minimum homozygous calling frequency of 0.99, and filtered out SNPs with low read coverage (coverage <8) (Koboldt *et al.* 2012). We used this coverage cutoff to reduce artifacts from sequencing and/or mapping error, and 8 was well below the coverage means for both of our datasets.

We also used our mapping data to generate an A619 variant file. We used Varscan (v. 0.1) to call SNPs in *ten* and 4 other A619 mutants. We did not assign a coverage cutoff for these datasets, and retained only SNPs that had a paired read. We used the function findCommonVariants in the Rsubread package (Shi *et al.* 2013) to identify SNPs common to all 5 datasets (File S1).

### Data processing for plotting and SNP filtering

Each filtered pileup file was split into 10 chromosomes. At each nucleotide position in the pileup file, we calculated variant allele frequency by dividing the number of reads that differed from the B73 reference sequence by the quality adjusted total number of reads at that position. This variant allele frequency was added as a new column in the pileup file. Nucleotide position, reference allele, alternate allele, coverage, and variant allele frequency were extracted from the pileup file to create a variant file for downstream analysis in R (Wickham and Francois 2015; Wickham 2016).

### Mapping region identification

We plotted our data against the B73 reference genome, to identify chromosomal regions enriched for homozygous SNPs, presumably in linkage with the causative lesions. Before plotting, we filtered positions with coverage >100 to remove highly enriched positions in our data. High coverage SNPs are likely in repeat rich regions and will not be informative for mapping region identification (Addo-Quaye *et al.* 2017). We used ggplot2 to plot the number of homozygous variant positions that differed from the B73 reference genome, per 1Mbp chromosomal bin (Wickham and Francois 2015; Wickham 2016). For these analyses, nucleotide positions that differed from the reference genome at a frequency greater than or equal to 0.99 were defined as homozygous variants. For *vns*, we went on to remove SNPs that were in the wild-type sibling and unmutagenized parent datasets, and plot only those homozygous SNPs that were unique to the mutant pool. For both *ten* and *vns*, we also plotted the number of homozygous canonical EMS variants (G to A and C to T transitions) per 1Mbp bin. We defined the limits of the *ten* and *vns* mapping intervals as those chromosomal coordinates where variant frequency returned to background levels.

### SNP filtering for candidate SNP identification

Once we had identified likely mapping intervals for *ten* and *vns*, we searched for potentially causative lesions in each of these intervals. Here, we returned to the vcf file generated using Varscan. Although we could have investigated the filtered EMS SNPs from our pileup file used to determine the mapping region directly, we chose to use Varscan because it outputs a vcf file, needed for downstream candidate SNP analyses using SnpEff (Cingolani *et al.* 2012). We filtered out all background A619 SNPs (File S1) from the *ten* dataset, and all parental and homozygous wild-type SNPs from the *vns* dataset. We reasoned that it was highly unlikely that the causative SNPs could be any known SNP present in any maize inbred. Therefore, we applied an additional SNP filtering step, and removed all the maize Hapmap 3.2.1 SNPs from both datasets, and our A619 SNPs from *vns* (Merchant *et al. 2016*; Bukowski *et al. 2018**)*. Once we had a set of positions enriched for SNPs unique to each of our particular mutant pools, we filtered out SNPs that were not homozygous and were not canonical EMS changes (G to A or C to T) (Till *et al.* 2004). The final set of homozygous EMS SNPs unique to each dataset was the input for an analysis of likely SNP effects on gene function.

To identify SNPs in our filtered datasets that might negatively affect gene function, we used SnpEff (version 4.3a). SnpEff identifies nonsense SNPs and splice site mutations as likely to have highly deleterious effects on gene function, and all missense SNPs as likely to have moderate effects on gene function (Cingolani *et al.* 2012). Functional annotations for the genes disrupted by likely moderate- and high-effect candidate SNPs were obtained from Gramene (Tello-Ruiz *et al.* 2018). Candidate SNPs were validated through Sanger sequencing and/or complementation crosses.

### Indel marker design and fine mapping

To refine our *ten* mapping interval, we designed custom markers for fine mapping. We used Varscan (v. 0.1) to identify insertion and deletion polymorphisms (indels) in our mapping interval that differentiated our mutant pool from the B73 reference genome. We chose indels that were 15 base pairs in length or longer, and designed primers to flank these indels by 100-150bp (Table S1). DNA was extracted from each of the 101 F2 mutants originally pooled into one NGS library and used for Indel PCR (Gallavotti and Whipple 2015). Size differences between the resulting PCR products were detected on 3.5% agarose gels.

### Data availability

Raw sequencing data available at NCBI SRA (https://www.ncbi.nlm.nih.gov/bioproject/PRJNA476333). A protocol for NGS data analysis and plotting in R is available at protocols.io (https://www.protocols.io/view/bsa-seq-in-maize-qyedxte). Supplemental material available at Figshare: https://doi.org/10.25387/g3.7014851.

## Results

### Mutant phenotypes

**Figure 3:**
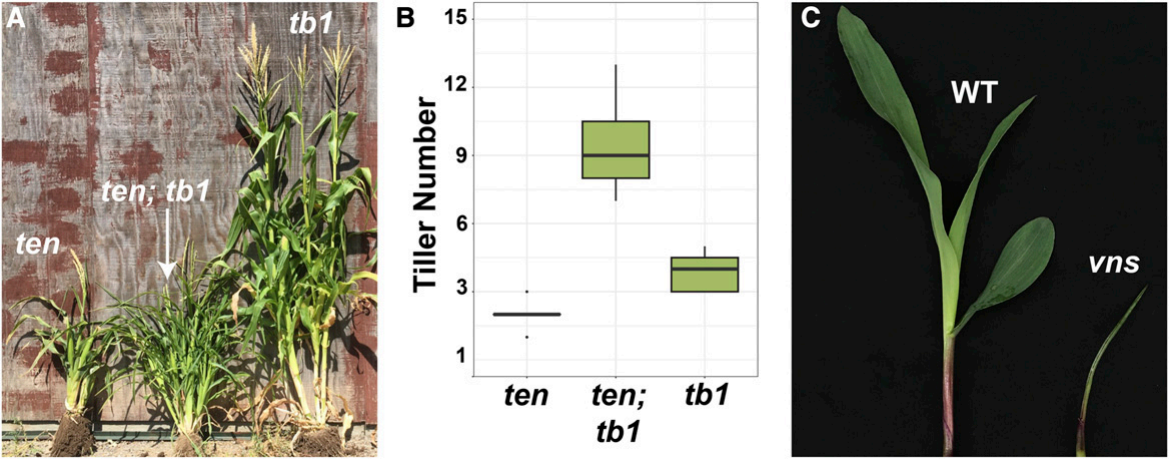
*ten* and *vns* were identified in EMS mutagenesis screens. (A) *ten* (left), *tb1-sh ten* (middle), *tb1-sh* (right). (B) Quantification of tillers in a 9:3:3:1 population of *ten* and *tb1-sh* shows a synergistic interaction between *ten* and *tb1-sh*. (C) Wild-type (left) and *vns* mutant (right) exhibiting reduced growth at the seedling stage.

From an EMS mutagenesis screen of *tb1-sh* mutants in the A619 genetic background, we recovered a dwarf mutant with additional tillers that we called *tb1 enhancer* (*ten*) (Figure 3a). *very narrow sheath* (*vns*) was uncovered in an independent EMS mutagenesis screen. *vns* single mutants are seedling lethal and often fail to develop more than 3 leaves (Figure 3c). Although *vns* was isolated in an *ns* enhancer screen, *vns* did not enhance *ns*.

To determine the nature of the genetic interaction between *ten* and *tb1*, we counted tillers in a population segregating both *tb1-sh* and *ten*. Tiller number (per plant) was counted in 15 *tb1-sh* single mutants, 15 *ten* single mutants and 15 *tb1-sh*; *ten* double mutants. *tb1-sh* and *ten* single mutants had an average of 4 and 2 tillers respectively. *ten tb1-sh* double mutants had an average of 9 tillers, showing a synergistic interaction between *ten* and *tb1-sh* in tiller development (Figure 3b).

### Both ten and vns are on chromosome one

To identify the causative lesions underlying the *ten* and *vns* mutant phenotypes, we sequenced pooled DNA from 101 *ten* individuals and 9 *vns* individuals from F2 mapping populations (Figure 1). We sequenced *ten* pools and *vns* pools on an Illumina platform with outputs of 125 bp and 150bp paired end reads, respectively. For *ten*, we recovered 450,665,452 reads, and for *vns* we recovered 311,711,046 reads. For *ten*, 97% of our reads (438,746,898) mapped to version 4 of the maize genome, and 91% were properly paired (409,548,662). For *vns*, 90% of our reads (279,239,524) mapped, and 77.5% were properly paired (241,580,812). The average coverage of all *ten* and *vns* mapped reads was 26-fold and 17-fold respectively (Table 1). The combined dataset that we used to generate the A619 SNP dataset included 1.1 billion mapped reads, for a mean coverage of 59-fold (File S1).

**Table 1:** Sequencing data summary for *ten* and *vns*

Measure	*ten*	*vns*
Reads	450,665,452	311,711,046
Mapped reads	438,746,898	279,239,524
% Mapped Reads	97.36	89.58
Paired reads	409,548,662	241,580,812
Coverage	26.06	16.59

**Figure 4:**
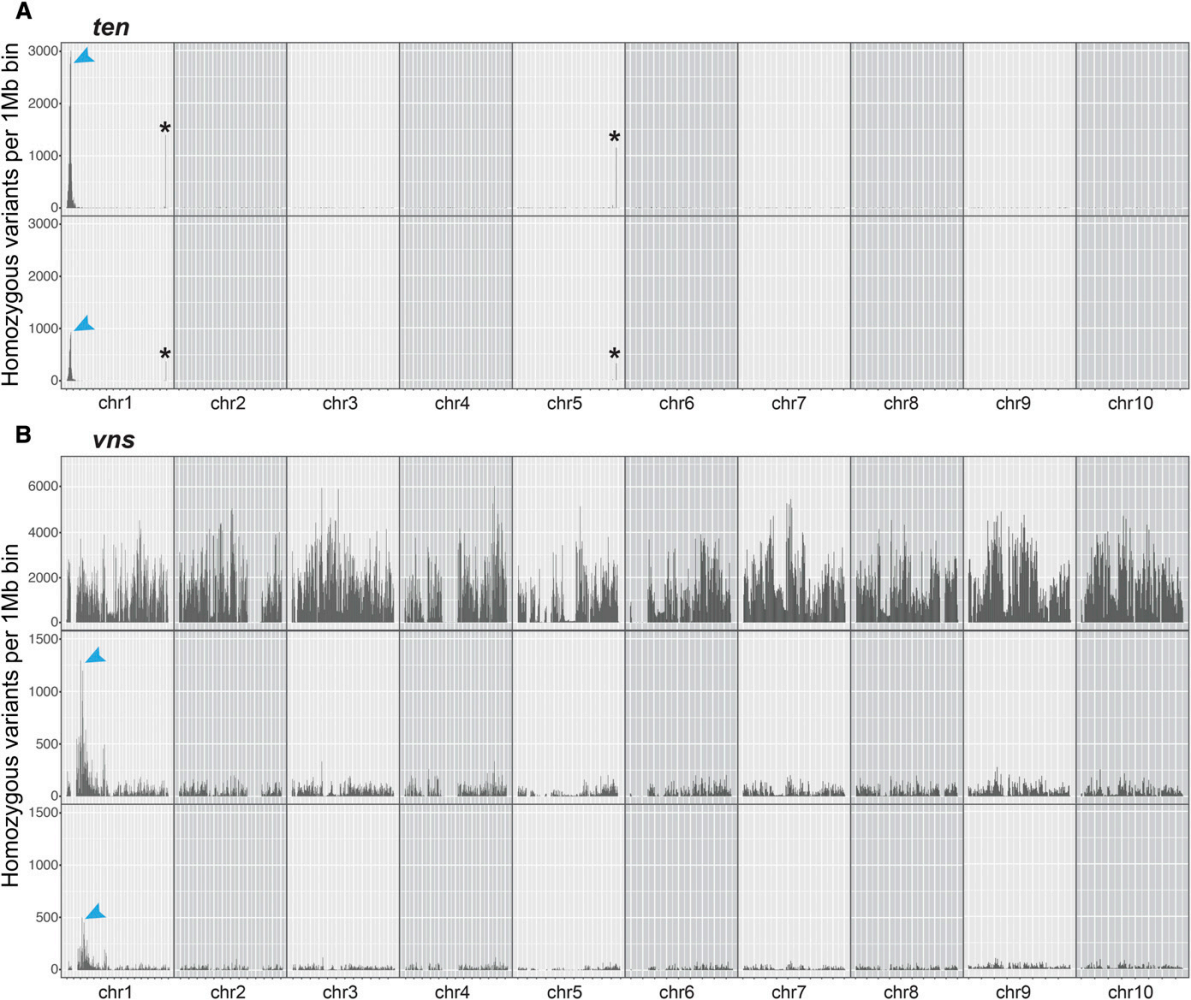
Both *ten* and *vns* are on chromosome 1. (A) *ten* likely lies between 0 and 20 Mbp on chromosome 1. Plotting the number of homozygous positions that differ from the B73 reference genome (per 1 Mbp chromosomal bin) reveals a large peak on chromosome 1 (top). This peak is still visible, but smaller, if only homozygous EMS SNPs are plotted (bottom). (B) *vns* likely lies between 40 and 70 Mbp on chromosome 1. Plotting the number of homozygous positions that differ from the B73 reference genome (per 1 Mbp chromosomal bin) reveals no distinct peaks (top). After filtering out parental, wild-type sibling, Hapmap 3.2.1 and background SNPs, a large peak on the short arm of chromosome 1 is revealed (middle). This peak is still visible, but smaller, if only homozygous EMS SNPs are plotted (bottom). *indicates a region where our B73 stocks likely differed from those that were used to generate the B73 reference genome.

To identify the chromosomal regions corresponding to *ten* and *vns*, we searched for regions of the genome enriched for homozygous variants in the mutant pools. For *ten*, we plotted the number of homozygous SNPs in the NGS data that differed from B73 (variant frequency >=0.99), per 1 Mbp bin. This plotting quickly identified a tall peak on the short arm of chromosome 1, between 0 and 20 Mbp. Plotting only homozygous canonical EMS SNPs (G to A and C to T transitions) identified a peak at the same position, although this peak was shorter (Figure 4a). We also identified two much smaller peaks at the telomeres of chromosome 1 and 5 that contained regions with protein coding genes. These telomeric peaks, at regions of the genome where recombination is high (Gore *et al.* 2009), were very narrow, and had sharp boundaries. In contrast, the peak at the top of chromosome 1 was much wider and taller than any of the telomeric peaks, and had broad shoulders. These broad shoulders are what we expected from a segregating locus mapped by BSA, where each genome in the pool will have different recombination breakpoints surrounding the mutant lesion. We hypothesized that the telomeric peaks were likely caused by differences between our B73 stocks and those used to generate the B73 reference genome. These differences may be because of the histories of our B73 stocks in our own labs, or because of the stocks’ provenance (Liang and Schnable 2016). We focused on the 0-20 Mbp peak at the top of chromosome 1 as the initial *ten* mapping interval, which included 623 genes.

For *vns*, identifying a chromosomal location was not quite as simple because *vns* arose in an unknown genetic background, and a *vns* heterozygote was crossed to an unmutagenized parent, and not outcrossed to any reference genotype (*e.g.*, B73) (Figure 1b). Thus, plotting only homozygous *vns* variants revealed, as expected, extensive homozygosity distinct from the B73 reference. There was still no clear peak when we plotted only canonical EMS SNPs. However, after removing all unmutagenized parental and wild-type sibling SNPs, as well as all the HapMap 3.2.1 and A619 SNPs, we found a region of high homozygosity on the short arm of chromosome 1 between 40 and 70 Mbp (Figure 4b). This *vns* interval was larger compared to the *ten* region, perhaps due to the small number of individuals (9) included in each *vns* pool, or the increased recombination at the end of chr.1 where *ten* is located. As with *ten*, plotting only EMS SNPs still revealed a (shorter) peak. The presence of many non-EMS SNPs in the *vns* mapping interval is likely a result of heterozygosity in the parental *ns1 ns2* stock. The *vns* mapping interval included 651 genes.

### Fine mapping of ten using custom indel markers quickly reduced the mapping interval

Both the *ten* and *vns* pools were sequenced fairly deeply (Table 1), and our sequencing data were thus likely to contain both causative lesions. However, in cases where mutant pools are sequenced to a shallower depth, and the causative lesions might be missed, we reasoned that the sequencing data could be used to design markers for fine mapping to reduce the mapping interval. This fine mapping will also be critical for reducing the mapping interval if there are multiple gene candidates in the mapping interval. Therefore, as a proof of concept, we used our NGS data to design five insertion/deletion (indel) markers to refine the *ten* interval (Gallavotti and Whipple 2015) (Table S1). We used these markers to genotype 101 *ten* individuals from the F2 mapping population and narrowed the *ten* interval to a region that includes 74 genes between 11.1 and 13.1 Mbp (Figure 4a). Thus, we were able to quickly reduce the *ten* mapping interval more than eightfold, and simplify the evaluation of candidate SNPs.

### SNP filtering and candidate SNP identification

Next, we wanted to identify candidate SNPs in our BSA-seq mapping regions. We used a variant calling program, Varscan, to call genotypes at variant positions based on allele frequency (Koboldt *et al.* 2012). Varscan identified 36,186,873 and 36,790,695 SNPs genome-wide in the *ten* and *vns* datasets, respectively (minimum read depth of 8) (Table 2). We removed putative background and non-causative SNPs from these datasets, as described in the Methods. After SNP filtering, we were left with 50 homozygous EMS SNPs in the *ten* mapping interval, and 427 homozygous EMS SNPs in the *vns* interval (allele frequency > 0.99).

**Table 2:** Varscan SNP data summary for *ten* and *vns* (coverage > 8)

SNPs	*ten* (ROI = 0–20Mbp)	*vns* (ROI = 40–70Mbp)
Genome-Wide		
Total SNPs (genome wide)	36,186,873	36,790,695
Homozygous EMS SNPs after filtering	148	7,899
Region of Interest (ROI)		
Total SNPs	266,837	470,763
Homozygous EMS SNPs after filtering	50	427
High effect homozygous EMS SNPs	0	1
Moderate effect homozygous EMS SNPs	4	2

To identify which SNPs in these final sets might have deleterious effects on gene function, we ran these SNPs through SnpEff (Cingolani *et al.* 2012). SnpEff identified 4 missense mutations in our *ten* mapping interval. The *vns* mapping interval included 1 splice site mutation, and 2 missense mutations (Table 3).

**Table 3:** Candidate SNPs for *ten* and *vns*

Chr	Position	Ref	Alt	Gene ID	SnpEff Call	Effect	Functional Annotation
*ten*							
1	12172055	C	T	Zm00001d027722	Moderate	L129F	*narrow odd dwarf (nod)*
1	12286537	C	T	Zm00001d027725	Moderate	P208L	3-deoxy-D-arabino-heptulosonate 7-phosphate synthase 1 (DHS1)
1	12631437	C	T	Zm00001d027752	Moderate	G139E	SH2 Domain Containing Protein
1	13337447	G	A	Zm00001d027775	Moderate	D67N	Histone H2A 8
*vns*							
1	47570165	G	A	Zm00001d028818	High	Splice Site	*defective kernel1* (*dek1*)
1	53173992	G	A	Zm00001d028965	Moderate	A38V	Protein of unknown function
1	53174101	G	A	Zm00001d028965	Moderate	R2C	Protein of unknown function

### Candidate SNP validation

In our *ten* dataset, the four missense SNPs were in genes encoding a 3-deoxy-D-arabino-heptulosonate 7-phosphate synthase 1 (*DHS1*) (Zm00001d027725); an SH2 domain protein (Zm00001d027752); Histone H2A 8 (Zm00001d027775); and a known maize gene - *narrow odd dwarf* (*nod*) (Zm00001d027722). *nod* encodes a maize MID-COMPLEMENTING ACTIVITY protein (Rosa *et al.* 2017). *nod-1* is a loss-of-function allele of *nod* in the B73 background (Rosa *et al.* 2017). Compared to the B73 inbred, *nod-1* exhibits several pleiotropic developmental defects, such as short stature, small organs, a lack of ligules, compromised apical dominance, and increased tiller growth (Rosa *et al.* 2017). This pleiotropic phenotype strongly resembled *ten* single mutants. Combined with our NGS data, this led us to speculate that *ten* could be an allele of *nod*.

**Figure 5:**
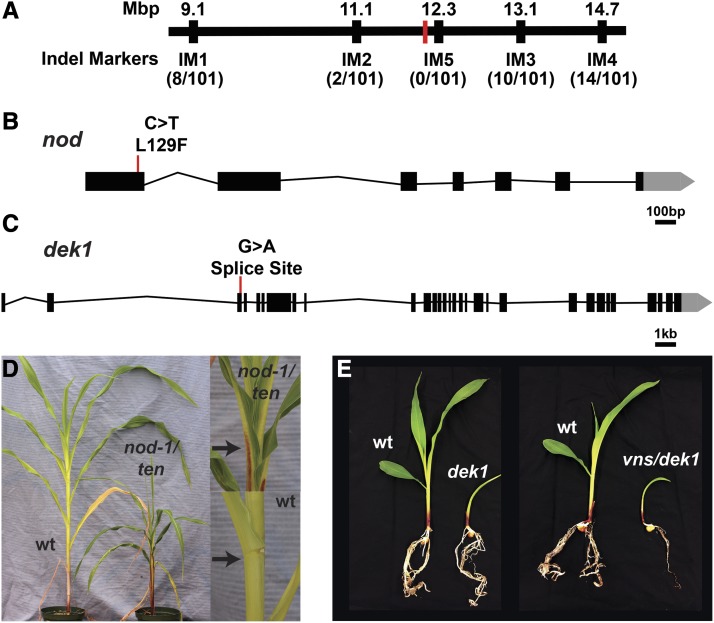
*ten* and *vns* encode alleles of *nod* and *dek1*, respectively. (A) Fine mapping of *ten* using custom indel markers reduced the mapping interval to a region containing 74 genes. The approximate position of *nod-ten* is marked in red. (B) The *ten* lesion in *nod* is a missense mutation. (C) The *vns* lesion in *dek1* is a splice site mutation. (D) *ten* fails to complement *nod*. As in *nod-1* mutants (Rosa *et al.* 2017), the ligule and auricle are both absent in the leaves of *ten/nod-1* F1 plants (top). The black arrow indicates the ligule in a wild-type plant (bottom), and the lack of a ligule in a *ten/nod-1* F1 plant. (E) *vns* fails to complement *dek1*.

To test whether *ten* was an allele of *nod*, we Sanger sequenced *nod* in one of our *ten* mutants, and performed complementation crosses. Sanger sequencing confirmed that *ten* harbored the same missense mutation in *nod* that was identified through NGS (Figure 5b). In our field, *nod-1* homozygous individuals failed to produce mature tassels or ears by the time that *ten* plants were ready for crossing. Therefore, we crossed *ten* to heterozygous *nod* plants and examined the F1 progeny. Both *ten* and *nod-1* are recessive mutations. Therefore, if *ten* were an allele of *nod*, we expected about half of the F1 progeny to show the *nod* phenotype, and half of the F1 progeny to show a wild-type phenotype. Otherwise, if *ten* were not an allele of *nod*, we expected all F1 progeny to show a wild-type phenotype. Indeed, we found that 12 of 26 F1 plants were short, and lacked ligules (*P* = 0.69, chi-squared test, one degree of freedom, Figure 5d), as is the case with *nod* mutants (Rosa *et al.* 2017). Therefore, we concluded that *ten* is an allele of *nod*, and henceforth will refer to it as *nod-ten*.

The single likely high effect SNP in the *vns* mapping interval was in a donor splice site of a known maize gene, *defective kernel1* (*dek1*) (Zm00001d028818) (Figure 5c). *Dek1* encodes a membrane-spanning protein with a calpain protease domain (Lid *et al.* 2002). *dek1* mutants contain a root primordium but lack a shoot structure (Neuffer *et al.* 1997), similar to what was observed in *vns* mutants. This led us to suspect that *vns* might encode an allele of *dek1*.

To determine if the SNP in *dek1* was responsible for the *vns* phenotype, we crossed heterozygous *vns* plants with heterozygous *dek1* plants. If *vns* were an allele of *dek1*, we expected about one quarter of the F1 progeny to show the *dek1* phenotype, and three quarters of the F1 progeny to show a wild-type phenotype. Otherwise, if *vns* were not an allele of *dek1*, we expected all F1 progeny to show a wild-type phenotype. In the F1 progeny, 9 of 36 plants were very small, and died shortly after producing only a small number of leaves (*P* = 1, chi-squared test, one degree of freedom, Figure 5e). Similarly, 7 of 36 F1 progeny of the heterozygous *dek1* self exhibited the same phenotype (*P* = 0.44, chi-squared test, one degree of freedom). Thus, *vns* failed to complement the *dek1* mutant phenotype, indicating that *vns* is an allele of *dek1*. Henceforth we will refer to *vns* as *dek1-vns*.

## Discussion

Here, we report a BSA-Seq method for cloning EMS mutants in maize. Using this method, we identified and validated causative SNPs for two separate EMS-induced mutants. We have provided a detailed protocol for conducting these analyses using Galaxy, as well as a SNP variant file for the A619 genetic background (File S1). This has the potential to be useful for anyone working in A619 - including for making a synthetic A619 reference genome (McKenna *et al.* 2010). In addition, we showed an interaction between the known maize genes *nod* and *tb1* (Doebley *et al.* 1997; Rosa *et al.* 2017).

We discovered a synergistic interaction between *tb1* and *nod* in regulating tiller production (Figure 3). Although *nod* is predicted to encode a membrane-localized maize MID-COMPLEMENTING ACTIVITY homolog, and may function to coordinate plant development in response to intrinsic and extrinsic cues (Rosa *et al.* 2017), how exactly *nod* regulates plant development remains largely unknown. The homolog of *NOD* in *Arabidopsis thaliana*, *MCA1*, may be involved in Ca^2+^ uptake and the cell wall stress response pathway (Yamanaka *et al.* 2010). In maize, hormone metabolism and cell division are overrepresented GO categories in *nod-1* mutant transcriptomes, relative to wild-type (Rosa *et al.* 2017). Interestingly, *tb1* is downregulated (2.4-fold) in *nod-1* shoot apices, relative to wild-type (Rosa *et al.* 2017), which suggests that *nod* might act upstream of *tb1* to regulate its expression. *nod* could represent a sensor of positional and environmental cues that regulate *tb1*-mediated tiller outgrowth. The exact mechanism through which *nod* and *tb1* synergistically regulate tiller outgrowth requires further investigation.

In any BSA-Seq experiment, the key goals are to define the smallest mapping interval possible, and to identify a small number of potentially causative lesions. To achieve these goals, both the size of the mutant pool and sequencing depth must be considered. In our experiments, the *nod-ten* BSA-Seq mapping interval encompassed ∼20Mbp on the short arm of chromosome 1, and included 623 genes. The *dek1-vns* mapping interval encompassed ∼30Mbp on the short arm of chromosome 1, and included 651 genes (Figure 4). Thus, despite a much smaller mutant pool (9 *dek1-vns* individuals *vs.* 101 *nod-ten* individuals), and a lower recombination rate where *dek1-vns* is located (Gore *et al.* 2009), the *dek1-vns* mapping interval included only 28 (4%) more genes than the *nod-ten* mapping interval. Mean coverage in both datasets was fairly high (26-fold for *ten* and 17-fold for *vns*), allowing for both causative lesions to be captured. To identify just the genomic interval that contains a particular gene, sequencing depth could be reduced, but the chances of catching the causative lesion will be similarly reduced. In a case where no clear candidate lesion is recovered, and/or there are many genes in a BSA-Seq mapping interval, fine mapping would become essential. Thus, our results indicate that sequencing to the deepest level affordable is more important than a big mutant pool for a successful BSA-Seq experiment.

The most important advantage of BSA-Seq over other methods is its simplicity, both in terms of sample collection and data analysis. While BSA-Seq is used extensively in other taxa (Schneeberger and Weigel 2011; Abe *et al.* 2012; Mascher *et al.* 2014; Woods *et al.* 2014; Ding *et al.* 2017; Song *et al.* 2017; Jiao *et al.* 2018), gene mapping via Bulked-Segregant RNA-Seq (BSR-Seq) is more often used for cloning maize genes (Liu *et al.* 2012; Li *et al.* 2013; Nestler *et al.* 2014; Tang *et al.* 2014). In BSR-Seq, RNA from a pool of mutants and RNA from a pool of non-mutants is used to make RNA-Seq libraries (Liu *et al.* 2012). These libraries are sequenced, and the resulting reads mapped to the maize reference genome. BSR-Seq offers a very good method for genome reduction, thus increasing sequencing depth without increasing cost. If the RNA-Seq is performed using the right tissue at the right developmental stage, BSR-Seq offers the advantage of differential gene expression data as well as mapping information. In contrast, since BSA-seq relies on DNA extraction, sample collection can be done at any developmental stage, and from any tissue. Although bulk RNA extractions are not extraordinarily challenging, DNA extractions are far simpler, and can be performed by relatively inexperienced trainees in the lab. This technical simplicity offers the advantage of being able to involve high school students and junior undergraduates in authentic research experiences (Lopatto *et al.* 2014). In addition, the data analysis for BSR-Seq is not as straightforward as it is for BSA-Seq. In BSR-Seq, allele-specific expression must be accounted for, as well as differential expression of genes not linked to the mutant gene in mutant *vs.* wild-type pools (Liu *et al.* 2012). Here, too, simplicity offers speed for the experienced researcher, and excellent training opportunities for beginning scientists.

For *dek1-vns*, we used a modification of BSA-Seq that has been called MutMap (Abe *et al.* 2012). In MutMap, instead of generating an F1 between contrasting genotypes, the F1 comes from a mutant backcrossed to a wild-type, unmutagenized individual in the same genetic background as the mutant. Thus, MutMap introduces no additional genetic variation in the initial F1 cross. Instead, MutMap relies on co-segregation of induced SNPs with the mutant phenotype, allowing for the identification of a region of increased homozygosity in the mutant pool. MutMap would likely also have been successful in identifying *nod-ten*. In both of our datasets, just mapping the EMS mutations reveals clear peaks corresponding to the mutant genes (Figure 4). One advantage that MutMap offers is that no additional genetic variation is introduced in the F1 cross. This genetic variation can suppress or enhance mutant phenotypes, which can make scoring F2 populations challenging. However, in these cases, conventional BSA-Seq could be used to identify both causative lesions and modifiers in one step (Song *et al.* 2017).

Another advantage of BSA-Seq over MutMap is that even if the lesion is not captured, or if there are many candidate lesions in a mapping interval, a researcher is immediately poised to design indel markers and commence fine-mapping in an identified genomic interval. Numerous candidate lesions are more likely when the mutant under study is not from an EMS mutagenesis experiment, which is fairly common in maize (Vollbrecht *et al.* 1991; Thompson *et al.* 2009; Whipple *et al.* 2011). In these cases, lesions are not necessarily of a defined type (*e.g.*, G to A or C to T transitions), which makes SNP filtering challenging. However, high homozygosity that is polymorphic with a reference genome will still reveal the chromosomal location of the causative lesion, and streamline subsequent fine mapping.

Technical and conceptual advances will eliminate some, but not all, sources of uncertainty when it comes to capturing the candidate lesions underlying mutant phenotypes. With shallow read depths, causative lesions may not be captured; but deep coverage is likely to become ever more attainable as NGS costs drop, thus eliminating this source of uncertainty. There may be a number of potentially causative lesions in a candidate region; but as the maize pan-genome is resolved (Hirsch *et al.* 2014; Bukowski *et al.* 2018), the pool of background SNPs that can be eliminated gets ever-deeper when cloning clear null mutations unlikely to be present in natural variation. Other potential sources of uncertainty will likely never go away. For example, filtering out all of the HapMap and pan-genome SNPs will be less useful when trying to identify natural modifiers. BSA-Seq and fine mapping will be particularly helpful in narrowing down a candidate region for a natural modifier; when a causative lesion is not in the coding sequence of an annotated gene; or when mutant pools are contaminated with wild-type or heterozygous samples. Thus, BSA-Seq offers many advantages that are likely to prove useful for mapping and cloning many maize mutants.

## References

[bib1] Abe A. Kosugi S. Yoshida K. Natsume S. Takagi H., 2012 Genome sequencing reveals agronomically important loci in rice using MutMap. Nat. Biotechnol. 30: 174–178. 10.1038/nbt.2095 22267009

[bib2] Addo-Quaye C. Buescher E. Best N. Chaikam V. Baxter I., 2017 Forward Genetics by Sequencing EMS Variation-Induced Inbred Lines. G3: Genes, Genomes. Genetics 7: 413–425. 10.1534/g3.116.029660 PMC5295590 28040779

[bib55] Afgan E. Baker D. Batut B. Van Den Beek M. Bouvier D. Čech M., 2018 The Galaxy platform for accessible, reproducible and collaborative biomedical analyses: 2018 update. Nuc. acids research, 46: W537–W544. 10.1093/nar/gky379 PMC6030816 29790989

[bib3] Afgan E. Baker D. van den Beek M. Blankenberg D. Bouvier D., 2016 The Galaxy platform for accessible, reproducible and collaborative biomedical analyses: 2016 update. Nucleic Acids Res. 44: W3–W10. 10.1093/nar/gkw343 27137889 PMC4987906

[bib4] Andrews S., 2014 FastQC: a quality control tool for high throughput sequence data. Version 0.11. 2. Babraham Institute, Cambridge, UK http://www. bioinformatics. babraham. ac.uk/projects/fastqc.

[bib5] Becraft P. W. Li K. Dey N. Asuncion-Crabb Y., 2002 The maize dek1 gene functions in embryonic pattern formation and cell fate specification. Development 129: 5217–5225. 12399313 10.1242/dev.129.22.5217

[bib6] Blankenberg D. Gordon A. Von Kuster G. Coraor N. Taylor J., 2010 Manipulation of FASTQ data with Galaxy. Bioinformatics 26: 1783–1785. 10.1093/bioinformatics/btq281 20562416 PMC2894519

[bib7] Bolger A. M. Lohse M. Usadel B., 2014 Trimmomatic: a flexible trimmer for Illumina sequence data. Bioinformatics 30: 2114–2120. 10.1093/bioinformatics/btu170 24695404 PMC4103590

[bib8] Bukowski R. Guo X. Lu Y. Zou C. He B., 2018 Construction of the third-generation Zea mays haplotype map. Gigascience 7: 1–12. 10.1093/gigascience/gix134 PMC5890452 29300887

[bib9] Cingolani P. Platts A. Wang L. L. Coon M. Nguyen T., 2012 A program for annotating and predicting the effects of single nucleotide polymorphisms, SnpEff: SNPs in the genome of Drosophila melanogaster strain w1118; iso-2; iso-3. Fly (Austin) 6: 80–92. 10.4161/fly.19695 22728672 PMC3679285

[bib10] Ding B. Mou F. Sun W. Chen S. Peng F., 2017 A dominant-negative actin mutation alters corolla tube width and pollinator visitation in Mimulus lewisii. New Phytol. 213: 1936–1944. 10.1111/nph.14281 28164332 PMC5300067

[bib11] Doebley J. Stec A. Hubbard L., 1997 The evolution of apical dominance in maize. Nature 386: 485–488. 10.1038/386485a0 9087405

[bib12] Ewing B. Hillier L. Wendl M. C. Green P., 1998 Base-Calling of Automated Sequencer Traces UsingPhred. I. Accuracy Assessment. Genome Res. 8: 175–185. 10.1101/gr.8.3.175 9521921

[bib13] Gallavotti A. Long J. A. Stanfield S. Yang X. Jackson D., 2010 The control of axillary meristem fate in the maize ramosa pathway. Development 137: 2849–2856. 10.1242/dev.051748 20699296 PMC2938917

[bib14] Gallavotti A. Whipple C. J., 2015 Positional cloning in maize (Zea mays subsp. mays, Poaceae). Appl. Plant Sci. 3: apps.1400092 10.3732/apps.1400092

[bib15] Gillmor C. S. Roeder A. H. K. Sieber P. Somerville C. Lukowitz W., 2016 A Genetic Screen for Mutations Affecting Cell Division in the Arabidopsis thaliana Embryo Identifies Seven Loci Required for Cytokinesis. PLoS One 11: e0146492 10.1371/journal.pone.0146492 26745275 PMC4712874

[bib16] Gore M. A. Chia J.-M. Elshire R. J. Sun Q. Ersoz E. S., 2009 A first-generation haplotype map of maize. Science 326: 1115–1117. 10.1126/science.1177837 19965431

[bib18] Haase N. J. Beissinger T. Hirsch C. N. Vaillancourt B. Deshpande S., 2015 Shared Genomic Regions Between Derivatives of a Large Segregating Population of Maize Identified Using Bulked Segregant Analysis Sequencing and Traditional Linkage Analysis. G3 (Bethesda) 5: 1593–1602. 10.1534/g3.115.017665 26038364 PMC4528316

[bib19] Hirsch C. N. Foerster J. M. Johnson J. M. Sekhon R. S. Muttoni G., 2014 Insights into the maize pan-genome and pan-transcriptome. Plant Cell 26: 121–135. 10.1105/tpc.113.119982 24488960 PMC3963563

[bib20] Jiao Y. Burow G. Gladman N. Acosta-Martinez V. Chen J., 2018 Efficient Identification of Causal Mutations through Sequencing of Bulked F2 from Two Allelic Bloomless Mutants of Sorghum bicolor. Front. Plant Sci. 8: 2267 10.3389/fpls.2017.02267 29379518 PMC5771210

[bib21] Jiao Y. Peluso P. Shi J. Liang T. Stitzer M. C., 2017 Improved maize reference genome with single-molecule technologies. Nature 546: 524–527. 10.1038/nature22971 28605751 PMC7052699

[bib22] Koboldt D. C. Zhang Q. Larson D. E. Shen D. McLellan M. D., 2012 VarScan 2: somatic mutation and copy number alteration discovery in cancer by exome sequencing. Genome Res. 22: 568–576. 10.1101/gr.129684.111 22300766 PMC3290792

[bib23] Langmead B. Salzberg S. L., 2012 Fast gapped-read alignment with Bowtie 2. Nat. Methods 9: 357–359. 10.1038/nmeth.1923 22388286 PMC3322381

[bib24] Liang Z. Schnable J. C., 2016 RNA-Seq Based Analysis of Population Structure within the Maize Inbred B73. PLoS One 11: e0157942 10.1371/journal.pone.0157942 27348435 PMC4922647

[bib25] Lid S. E. Gruis D. Jung R. Lorentzen J. A. Ananiev E., 2002 The defective kernel 1 (dek1) gene required for aleurone cell development in the endosperm of maize grains encodes a membrane protein of the calpain gene superfamily. Proc. Natl. Acad. Sci. USA 99: 5460–5465. 10.1073/pnas.042098799 11929961 PMC122791

[bib26] Li H. Handsaker B. Wysoker A. Fennell T. Ruan J., 2009 The Sequence Alignment/Map format and SAMtools. Bioinformatics 25: 2078–2079. 10.1093/bioinformatics/btp352 19505943 PMC2723002

[bib27] Li L. Li D. Liu S. Ma X. Dietrich C. R., 2013 The maize glossy13 gene, cloned via BSR-Seq and Seq-walking encodes a putative ABC transporter required for the normal accumulation of epicuticular *PLoS One* 8: e82333 10.1371/journal.pone.0082333 PMC3855708 24324772

[bib56] Liao Y. Smyth G. K. Shi W., 2013 The Subread aligner: fast, accurate and scalable read mapping by seed-and-vote. Nuc. Acids Res., 41: e108. 10.1093/nar/gkt214 PMC3664803 23558742

[bib28] Liu S. Yeh C.-T. Tang H. M. Nettleton D. Schnable P. S., 2012 Gene Mapping via Bulked Segregant RNA-Seq (BSR-Seq). PLoS One 7: e36406 10.1371/journal.pone.0036406 22586469 PMC3346754

[bib29] Lopatto D. Hauser C. Jones C. J. Paetkau D. Chandrasekaran V., 2014 A central support system can facilitate implementation and sustainability of a Classroom-based Undergraduate Research Experience (CURE) in Genomics. CBE Life Sci. Educ. 13: 711–723. 10.1187/cbe.13-10-0200 25452493 PMC4255357

[bib30] Mascher M. Jost M. Kuon J.-E. Himmelbach A. Aßfalg A., 2014 Mapping-by-sequencing accelerates forward genetics in barley. Genome Biol. 15: R78 10.1186/gb-2014-15-6-r78 24917130 PMC4073093

[bib31] McKenna A. Hanna M. Banks E. Sivachenko A. Cibulskis K., 2010 The Genome Analysis Toolkit: a MapReduce framework for analyzing next-generation DNA sequencing data. Genome Res. 20: 1297–1303. 10.1101/gr.107524.110 20644199 PMC2928508

[bib32] Merchant N. Lyons E. Goff S. Vaughn M. Ware D., 2016 The iPlant Collaborative: Cyberinfrastructure for Enabling Data to Discovery for the Life Sciences. PLoS Biol. 14: e1002342 10.1371/journal.pbio.1002342 26752627 PMC4709069

[bib33] Michelmore R. W. Paran I. Kesseli R. V., 1991 Identification of markers linked to disease-resistance genes by bulked segregant analysis: a rapid method to detect markers in specific genomic regions by using segregating populations. Proc. Natl. Acad. Sci. USA 88: 9828–9832. 10.1073/pnas.88.21.9828 1682921 PMC52814

[bib34] Mueller R. J., 2006 Ask the plant: investigating and teaching plant structure. Bot. J. Linn. Soc. 150: 73–78. 10.1111/j.1095-8339.2006.00489.x

[bib35] Nardmann J. Ji J. Werr W. Scanlon M. J., 2004 The maize duplicate genes narrow sheath1 and narrow sheath2 encode a conserved homeobox gene function in a lateral domain of shoot apical meristems. Development 131: 2827–2839. 10.1242/dev.01164 15169755

[bib36] Nawy T. Bayer M. Mravec J. Friml J. Birnbaum K. D., 2010 The GATA factor HANABA TARANU is required to position the proembryo boundary in the early Arabidopsis embryo. Dev. Cell 19: 103–113. 10.1016/j.devcel.2010.06.004 20643354

[bib37] Nestler J. Liu S. Wen T.-J. Paschold A. Marcon C., 2014 Roothairless5, which functions in maize (Zea mays L.) root hair initiation and elongation encodes a monocot-specific NADPH oxidase. Plant J. 79: 729–740. 10.1111/tpj.12578 24902980

[bib38] Neuffer M. G. Coe E. H. Wessler S. R., 1997 *Mutants of maize*. Cold Spring Harbor Laboratory Press, New York.

[bib39] Rosa M. Abraham-Juárez M. J. Lewis M. W. Fonseca J. P. Tian W., 2017 The Maize MID-COMPLEMENTING ACTIVITY Homolog CELL NUMBER REGULATOR13/NARROW ODD DWARF Coordinates Organ Growth and Tissue Patterning. Plant Cell 29: 474–490. 10.1105/tpc.16.00878 28254777 PMC5385958

[bib40] Scanlon M. J. Schneeberger R. G. Freeling M., 1996 The maize mutant narrow sheath fails to establish leaf margin identity in a meristematic domain. Development 122: 1683–1691. 8674408 10.1242/dev.122.6.1683

[bib41] Schneeberger K. Weigel D., 2011 Fast-forward genetics enabled by new sequencing technologies. Trends Plant Sci. 16: 282–288. 10.1016/j.tplants.2011.02.006 21439889

[bib43] Song J. Li Z. Liu Z. Guo Y. Qiu L.-J., 2017 Next-Generation Sequencing from Bulked-Segregant Analysis Accelerates the Simultaneous Identification of Two Qualitative Genes in Soybean. Front. Plant Sci. 8: 919 10.3389/fpls.2017.00919 28620406 PMC5449466

[bib44] Tang H. M. Liu S. Hill-Skinner S. Wu W. Reed D., 2014 The maize brown midrib2 (bm2) gene encodes a methylenetetrahydrofolate reductase that contributes to lignin accumulation. Plant J. 77: 380–392. 10.1111/tpj.12394 24286468 PMC4282534

[bib45] Tello-Ruiz M. K. Naithani S. Stein J. C. Gupta P. Campbell M., 2018 Gramene 2018: unifying comparative genomics and pathway resources for plant research. Nucleic Acids Res. 46: D1181–D1189. 10.1093/nar/gkx1111 29165610 PMC5753211

[bib46] Thompson B. E. Bartling L. Whipple C. Hall D. H. Sakai H., 2009 bearded-ear Encodes a MADS Box Transcription Factor Critical for Maize Floral Development. The Plant Cell Online 21: 2578–2590. 10.1105/tpc.109.067751 PMC2768933 19749152

[bib47] Till B. J. Reynolds S. H. Weil C. Springer N. Burtner C., 2004 Discovery of induced point mutations in maize genes by TILLING. BMC Plant Biol. 4: 12 10.1186/1471-2229-4-12 15282033 PMC512284

[bib48] Vlad D. Kierzkowski D. Rast M. I. Vuolo F. Dello Ioio R., 2014 Leaf Shape Evolution Through Duplication, Regulatory Diversification, and Loss of a Homeobox Gene. Science 343: 780–783. 10.1126/science.1248384 24531971

[bib49] Vollbrecht E. Veit B. Sinha N. Hake S., 1991 The developmental gene Knotted-1 is a member of a maize homeobox gene family. Nature 350: 241–243. 10.1038/350241a0 1672445

[bib50] Whipple C. J. Kebrom T. H. Weber A. L. Yang F. Hall D., 2011 grassy tillers1 promotes apical dominance in maize and responds to shade signals in the grasses. Proc. Natl. Acad. Sci. USA 108: E506–E512. 10.1073/pnas.1102819108 21808030 PMC3158142

[bib51] Wickham H., 2016 ggplot2: Elegant Graphics for Data Analysis. Springer-Verlag, New York.

[bib52] Wickham H. Francois R., 2015 dplyr: A grammar of data manipulation. R package version 0. 4 3.

[bib53] Woods D. P. Ream T. S. Minevich G. Hobert O. Amasino R. M., 2014 PHYTOCHROME C is an essential light receptor for photoperiodic flowering in the temperate grass, Brachypodium distachyon. Genetics 198: 397–408. 10.1534/genetics.114.166785 25023399 PMC4174950

[bib57] Yamanaka T. Nakagawa Y. Mori K. Nakano M. Imamura T., 2010 MCA1 and MCA2 that mediate Ca2+ uptake have distinct and overlapping roles in Arabidopsis. Plant Physio., 152: 1284–1296. 10.1104/pp.109.147371 PMC2832256 20097794

[bib54] Zou C. Wang P. Xu Y., 2016 Bulked sample analysis in genetics, genomics and crop improvement. Plant Biotechnol. J. 14: 1941–1955. 10.1111/pbi.12559 26990124 PMC5043468

